# The association of semaphorins 3C, 5A and 6D with liver fibrosis stage in chronic hepatitis C

**DOI:** 10.1371/journal.pone.0209481

**Published:** 2018-12-28

**Authors:** Neven Papic, Snjezana Zidovec Lepej, Lana Gorenec, Ivana Grgic, Slavko Gasparov, Tajana Filipec Kanizaj, Adriana Vince

**Affiliations:** 1 Department of Viral Hepatitis, University Hospital for Infectious Diseases Zagreb, Zagreb, Croatia; 2 University of Zagreb, School of Medicine, Zagreb, Croatia; 3 Department of Immunological and Molecular Diagnostics, University Hospital for Infectious Diseases, Zagreb, Croatia; 4 Department of Pathology and Cytology, University Hospital Merkur, Zagreb, Croatia; 5 Department of Gastroenterology, University Hospital Merkur, Zagreb, Croatia; University of Cincinnati College of Medicine, UNITED STATES

## Abstract

Semaphorins are a diverse family of immunoregulators recently recognized to play a major role in various phases of immune responses. Their role in chronic viral hepatitis C (CHC) and contribution to the progression of liver disease is unknown. The aim of this study was to analyse the association of secreted semaphorins with the severity of liver disease in patients with CHC. Serum concentrations of semaphorins were measured in 114 treatment-naive CHC patients and 36 healthy controls. Serum concentrations of SEMA3A, SEMA3C, SEMA5A, SEMA6B and SEMA6D were significantly increased in patients with CHC compared to controls. While serum concentrations of SEMA3C and SEMA6D significantly increased with fibrosis stage in both HCV-g1 and HCV-g3 infections, the concentration of SEMA5A inversely correlated with fibrosis stage in both HCV genotypes. ROC analysis showed that serum concentrations of SEMA3C (>4.0ng/mL, AUC 0.88) and SEMA6D (>4.5, AUC 0.82) had higher AUC than widely used APRI (AUC 0.71) and FIB-4 (AUC 0.74) scores. Serum concentrations of SEMA3C and SEMA6D significantly decreased after DAA and PEG IFN-α/ribavirin therapy, while the serum concentration of SEMA5A significantly increased after DAAs therapy. Immunohistochemistry confirmed the expression of SEMA3C and SEMA5A in hepatocytes, endothelial cells and lymphocytes of cirrhotic livers from CHC patients but not in controls. In conclusion, we provide the first evidence that SEMA3C, SEMA5A and SEMA6D can be considered as markers of liver injury in CHC.

## Introduction

Since its discovery 30 years ago, chronic hepatitis C (CHC) has evolved from an incurable disease to the prototype of chronic viral infection that can be easily and safely cured. Still, chronic hepatitis C remains the leading cause of cirrhosis, hepatocellular carcinoma and liver transplantation [1, 2]. Thus, the scientific focus in CHC research is moving rapidly from antiviral treatment towards complications of chronic infection and its early identification. Now we are able to eliminate the virus, but significant gaps in the knowledge of biological and immunological mechanisms of chronic infection, fibrogenesis and carcinogenesis remain.

System biology approaches and whole genome sequencing analyses have enabled us to identify molecular signatures that might be associated with the lack of effective immune responses, development of tolerance and low level inflammation associated with persistent HCV infection [3–9]. In transcriptome studies of *in vitro* HCV infected liver non-parenchymal cells, the semaphorin pathway emerged as a novel consistent finding associated with HCV infection [5–7, 10].

Semaphorins are a large family of secreted and membrane-bound biological response modifiers present in many organ systems, including the nervous, epithelial, and immune system that mediate the development, angiogenesis and oncogenic transformation [11–13]. A recent research describes the role of selected semaphorins in cellular immunity and subsequent levels of inflammatory response [12, 13]. While some suppress the immune cell activation, proliferation and reduce the production of pro-inflammatory cytokines, others stimulate the immune-mediated responses [12, 13]. The importance of plexins and semaphorins has been further emphasized in allergic and immune-mediated disorders, such as systemic lupus erythematosus (SLE), asthma, psoriasis, and multiple sclerosis [14]. So far, semaphorins have not been studied in HCV infected patients.

Based on the potential correlation between semaphorins`expression and disease progression, we hypothesized that semaphorins are associated with liver fibrosis resulting from chronic viral hepatitis and therefore may be a novel biomarker of liver cirrhosis. In the present study we investigated the expression profiles of selected semaphorins in the sera and liver samples from HCV infected patients, their correlations with clinicopathological data and treatment outcomes. The potential usefulness as biomarkers for early diagnosis of liver cirrhosis was also assessed.

## Materials and methods

### Patients and samples

The prospective study included 114 treatment-naive adult patients with chronic hepatitis C receiving clinical care at the Croatian Reference Center for Viral Hepatitis, Zagreb, Croatia between January 2016 and September 2018. All patients tested negative for HIV and HBV infection. Additionally, paired samples before and 12 weeks after antiviral therapy were analyzed in 23 patients (14 treated with direct acting antivirals without interferon, and 9 patients treated with pegylated interferon alpha) that achieved sustained virological response (SVR, defined as undetectable HCV RNA at 12 weeks after the end of antiviral therapy). 36 voluntary blood donors without relevant medical history and without obesity, the use of medication and/or alcohol abuse were included as healthy controls (HC).

The patients were enrolled consecutively upon signing the informed consent. Ethical approval was obtained from the Ethics Committee at the University Hospital for Infectious Diseases Zagreb, Croatia, in accordance with the declaration of Helsinki.

Whole blood samples were collected by venipuncture into sterile tubes without anticoagulant and the blood was allowed to clot for 20 minutes at room temperature. Serum was separated by centrifugation at 2000xg for 10 min. The samples were divided into aliquots to avoid repeated freeze and thaw cycles and stored at -80°C until testing.

Additionally, immunohistochemistry was performed on specimens obtained from 6 patients at the Liver Transplant Center (University Hospital Merkur, Zagreb, Croatia). Three specimens were obtained from healthy liver donors immediately after the explantation and three from the explanted cirrhotic CHC livers.

### Methods

The routine workup included complete blood count, liver function tests, HCV and HBV markers and HIV Ag/Ab, which were performed using commercially available assays. HCV RNA was quantified by using the standardized real-time PCR assay Abbott Real Time HCV test (Abbott Molecular, Illinois, USA) with a lower limit of detection of 12 IU/mL.

HCV genotypes were determined by using the Versant HCV genotype 2.0 Assay (Siemens Healthcare Diagnostics, Erlangen, Germany). The stage of liver fibrosis was assessed by transient elastography (Fibroscan, Echosens, Paris, France) [15]. APRI and FIB-4 score were calculated for all patients and were used as a surrogate marker of disease severity [16, 17]. The quantification of semaphorins was performed by using the standardized ELISA assays (Human Semaphorin -3A, -4D, -3C, -5A, -6B and -6D ELISA Kit, MyBioSource, San Diego, CA, USA), as recommended by the manufacturer. Quantification ranges of semaphorin ELISAs were as follows: SEMA-3A (31,25–2000 pg/ml), SEMA-4D (0,625–20 ng/ml), SEMA-3C 0–10 ng/ml, SEMA-5A (0,78–50 ng/ml), SEMA-6B (0,625–20 ng/ml) and SEMA-6D (0,625–20 ng/ml).“

Immunohistochemical staining: unstained slides (4 μm thick) were generated from the whole tissue blocks (2 blocks per case) for immunohistochemical staining using antibodies SEMA3C and SEMA5A. Immunohistochemical staining was performed in the automated immunostainer (Dako Autostainer, LINK 48, Denmark) using Envision (DAKO Denmark) for SEMA3C and ImmPRESS (Vector laboratories, USA) polymer detection systems for SEMA5A according to the manufacturer's instructions. Rabbit polyclonal anti-semaphorin 3C antibody (Abcam, Cambridge, UK) was used at 1:50 dilution. Goat polyclonal anti-semaphorin 5A antibody (Abcam, Cambridge, UK) was used at 1:80 dilution. Human pancreas tissue was used as a positive control for SEMA3C expression and human colon tissue as a positive control for SEMA5A expression (both cytoplasmic staining). For the negative control, adjacent tissue sections were stained without the primary antibody.

### Statistical analysis

The demographic, clinical characteristics and laboratory data were evaluated and presented descriptively. Categorical data were expressed in frequencies and relevant percentage. The significance of the observed differences in frequencies between relevant subgroups was tested with the chi-square test or the Fisher’s exact test when appropriate. For continuous variables, we calculated mean values with standard deviations or median values with 25th and 75th percentile depending on distribution. Comparative statistics between two or more groups was generated using the Mann–Whitney U or the Kruskal–Wallis test, as appropriate. Correlations were analyzed using the Spearman’s rank correlation coefficient. The diagnostic performances of the laboratory variables considered were compared using a receiver operating characteristic (ROC) analysis. Sensitivity, specificity, positive and negative predictive values and likelihood ratios (LRs) were calculated. All tests were two-tailed; a P < 0.05 was considered statistically significant. Statistical analyses were performed using prism (ver 5.0) statistical software (Graphpad Software, San Diego, CA) and MedCalc for Windows, ver 11.5.1.0 (MedCalc Software, Mariakerke, Belgium).

## Results

### Patient characteristics

The characteristics of the patients are shown in Table 1. The majority of CHC patients were infected with HCV genotype 1 (n = 70) and 44 patients were infected with genotype 3. CHC patients were classified into four groups according to the transient elastography (F1 n = 22; F2 n = 22, F3 n = 28, F4 n = 42). The study also included 36 healthy controls (19 males, median age of 51 years (IQR 39–59.7).

**Table 1:** Baseline patient characteristics.

	Fibrosis stage			
	1	2	3	4
Liver stiffness, kPa	5.5 (4.6–6.1)	7.9 (7.3–8.8)	10.2 (9.6–11)	19.2 (14.0–30.7)
Age, years	51.5 (43.7–65.5)	57 (51.75–65.25)	59.5 (41.5–66.7)	59 (48.7–63)
Male sex	10 (8.8%)	12 (10.5%)	12 (10.5%)	22 (19.3%)
HCV genotype 1	12 (10.5%)	14 (12.3%)	18 (15.8%)	26 (22.8%)
HCV genotype 3	10 (8.8%)	8 (7.0%)	10 (8.8%)	16 (14.0%)
HCV RNA, log 10	5.1 (4.9–5.4)	6.3 (5.2–6.7)	5.5 (4.5–6.1)	5.8 (5.2–6.1)
Aspartate transaminase, IU/L	45 (34.5–87.5)	61.5 (47–129)	79 (61.2–89)	82 (60.5–101.3)
Alanine transaminase, IU/L	67.5 (39.2–124.5)	77 (58.7–148)	97 (68–124.8)	92 (57–127.5)
Platelets, x10^9^/L	230 (184–300)	202 (147–231)	131.5 (98.2–194.3)	122.5 (78–220.5)
Albumins, g/L	43 (38.3–45.3)	42 (37.5–44.7)	38.5 (35–42.0)	34.4 (26.7–40.8)
International normalized ratio	1 (0.99–1.12)	1.0 (0.9–1.1)	1.13 (1.0–1.3)	1.3 (1.1–1.8)
APRI score	0.53 (0.37–0.68)	0.93 (0.58–1.72)	1.69 (0.85–2.5)	1.57 (0.82–3.01)
FIB4 score	1.21 (0.89–2.14)	2.3 (1.5–4.2)	3.08 (2.08–5.19)	3.31 (2.05–6.35)

Presented are baseline patient clinical, laboratory and virological characteristics according to fibrosis stage as measured by transient elastography. Data are presented as frequencies (%) or medians with interquartile ranges.

### Elevated serum concentrations of semaphorins in patients with chronic hepatitis C

**Fig 1:**
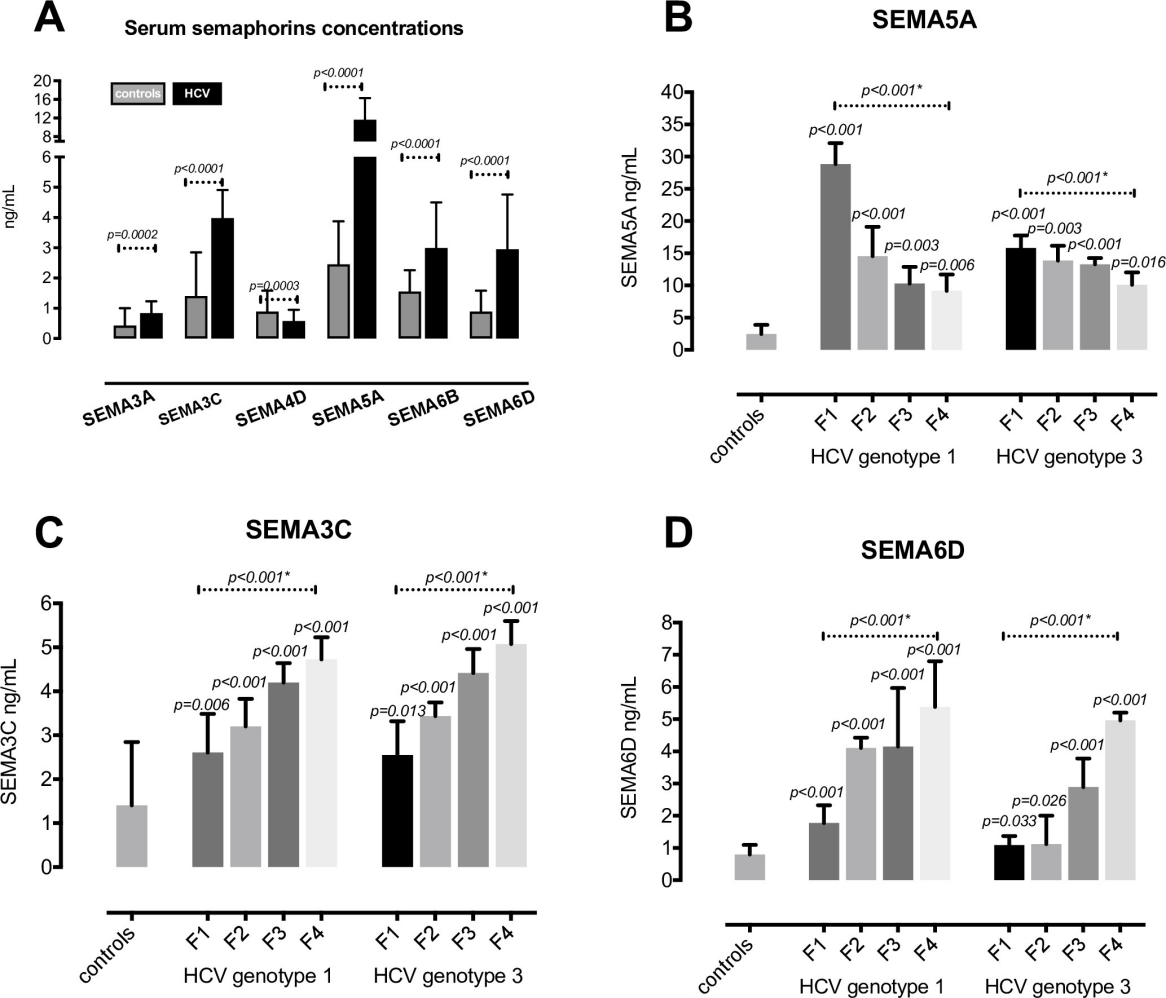
Serum concentrations of semaphorins in patients with chronic hepatitis C (CHC) and healthy controls. (**A**) Patients with CHC have elevated serum levels of semaphorins compared to healthy controls. Serum concentration of SEMA5A decreases with fibrosis progression (**B**), while serum concentrations of SEMA3C (**C**) and SEMA6D (**D**) increase with fibrosis stage. Data are presented as median with interquartile range and analyzed by Mann-Whitney *U*-test or Kruskal–Wallis test, as appropriate.

We examined serum semaphorin concentrations in CHC group compared to healthy controls. In comparison to healthy controls, serum levels of SEMA3A, SEMA3C, SEMA5A, SEMA6B and SEMA6D were significantly higher in CHC patients, whereas only SEMA4D was not significantly altered, as shown in Fig 1A. There was no difference in semaphorin expression in healthy controls regarding sex and age.

### Serum concentrations of SEMA3C, SEMA5A and SEMA6D correlate with fibrosis stage

Next, we analyzed serum concentrations of semaphorins in different fibrosis stages and HCV genotype infections (Fig 1B–1D). While serum concentrations of SEMA3C and SEMA6D significantly increased with fibrosis stage in both HCV-g1 and HCV-g3 infection, serum concentration of SEMA5A inversely correlated with fibrosis stage in both HCV genotypes. SEMA3A and SEMA6B serum concentrations showed no correlation with fibrosis stage.

In CHC patients, both SEMA3C and SEMA6D showed correlation with the APRI (r = 0.38, p<0.0001 and r = 0.36, p<0.0001, respectively) and FIB4 score (r = 0.39, p<0.0001 and r = 0.37, p<0.0001). SEMA3C and SEMA6D showed positive correlation with AST (r = 0.25, p = 0.0052 and r = 0.25, p = 0.0047, respectively), and negative correlation with platelet count (r = -0.35, p<0.0001 and r = -0.33, p = 0.0003). SEMA5A inversely correlated with AST (r = -0.23, p = 0.0125) and APRI (r = -0.36, p<0.0001) and FIB4 (r = -0.37, p<0.0001), and positively with platelet count (r = 0.35, p = 0.0001). Neither SEMA3C, SEMA6D nor SEMA5A correlated with age, viraemia and ALT.

### Serum SEMA3C and SEMA6D are superior to APRI and FIB-4 in predicting the presence of cirrhosis and significant fibrosis

Based on the above-mentioned correlations, the diagnostic performances of the selected semaphorins were compared to routinely used APRI [16] and FIB-4 [17] scores using a receiver operating characteristic (ROC) analysis.

**Fig 2:**
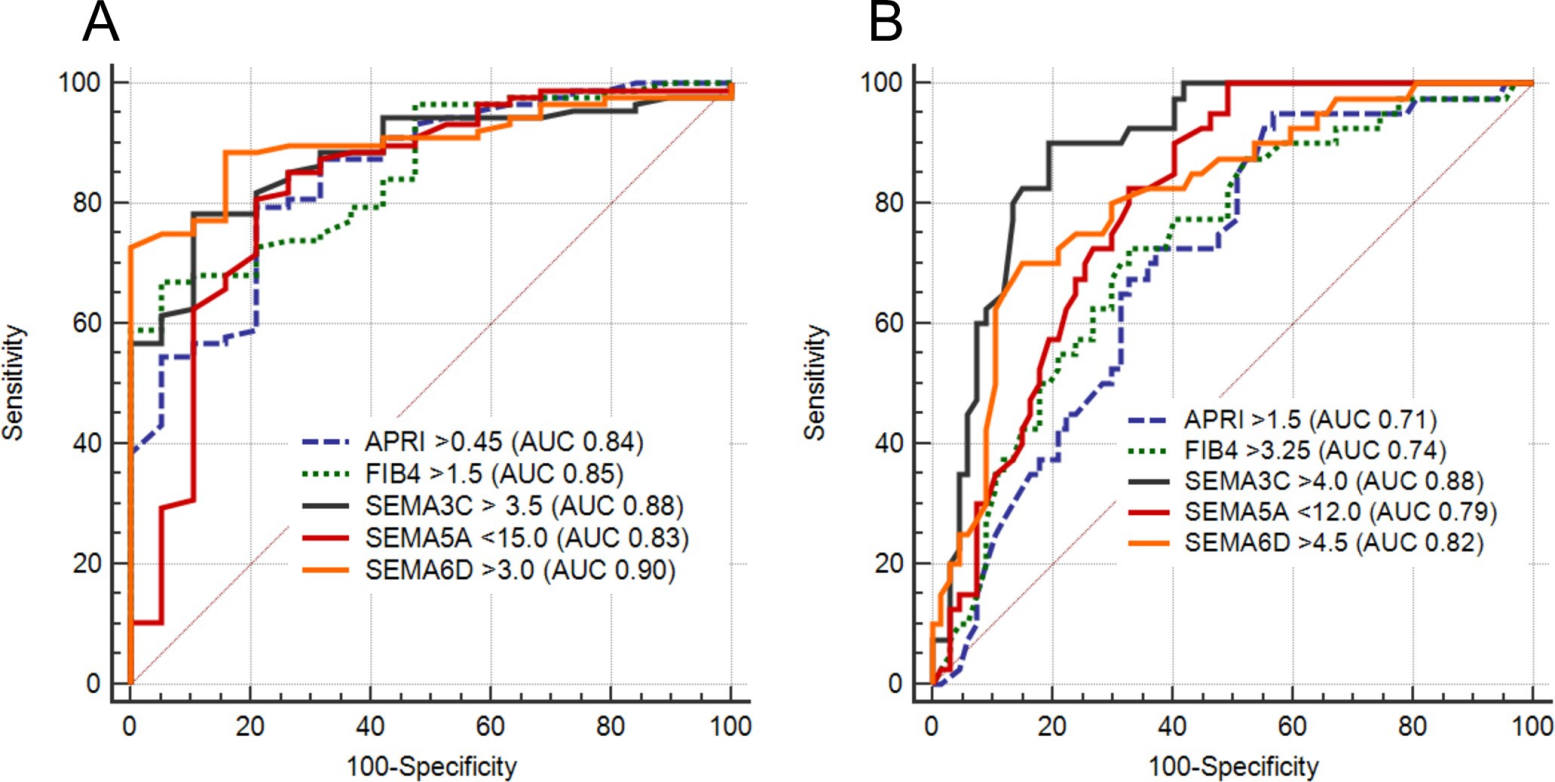
**The ROC curve of laboratory parameters for discrimination of liver cirrhosis (F4) (A) and significant liver fibrosis (F2-4) (B).** Shown are calculated cut-off values (Youden index), accuracy as measured by the area under the ROC curve (AUC) and probability as calculated p-values.

Firstly, serum concentrations of SEMA3C and SEMA6D showed better sensitivity and specificity for detection of liver cirrhosis (>4.0, sensitivity 87.5%, specificity 78.8%, AUC 0.88 and >4.5, sensitivity 72.9%, specificity 85.7%, AUC 0.82, respectively) as compared to widely used APRI score (>1.4 sensitivity 68.7%, specificity 69.1%, AUC 0.71) or FIB-4 score (>3.25 sensitivity 64.5%, specificity 70.4%, AUC 0.74), as shown in Fig 2A. Serum concentration of SEMA5A <12ng/mL showed sensitivity of 85.0% and specificity of 63.3% (AUC 0.79) in the detection of cirrhosis.

Secondly, we examined the diagnostic accuracy in detection of significant fibrosis (F2-F4). Again, SEMA3C >3.5 (sensitivity 78.3%, specificity 89.4%, AUC 0.88), SEMA6D >3.0 (sensitivity 77.0%, specificity 95.4%, AUC 0.90) and SEMA5A <15 (sensitivity 85.3%, specificity 72.3%, AUC 0.83) showed comparable discrimination of significant fibrosis as did APRI (AUC 0.84) and FIB4 (AUC 0.85) scores, as shown in Fig 2B.

### Serum levels of SEMA3C and SEMA6D decrease after DAA and PegIFNα treatment

**Fig 3:**
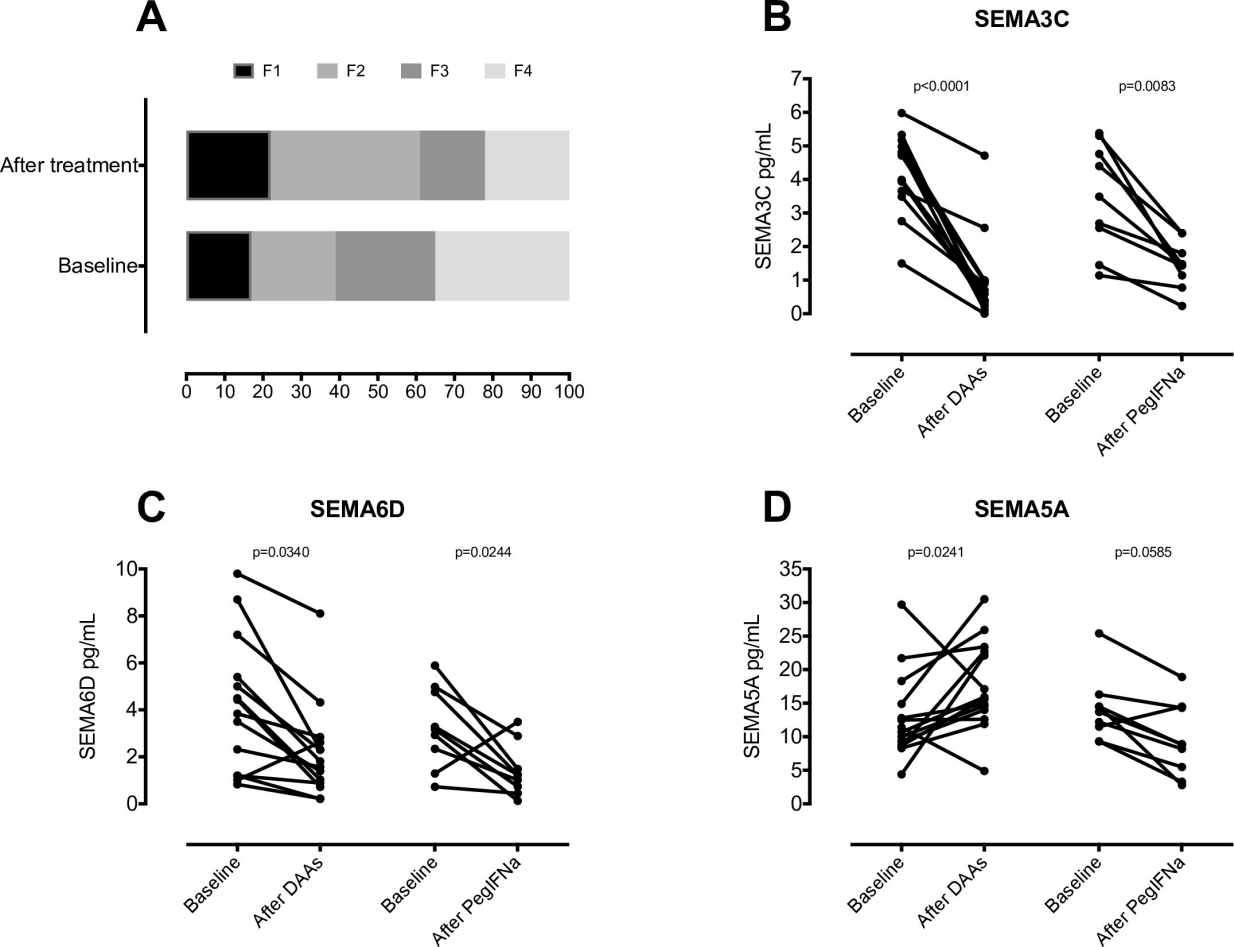
Serum semaphorins levels and liver fibrosis in patients before and after antiviral therapy. Changes in the proportion of fibrosis (F1-4) (**A**), serum SEMA3C (**B**), SEMA6D (**C**) and SEMA5A (**D**) in patients before and after antiviral therapy are shown. Paired patient concentrations are shown (n = 23). Data were analyzed by Mann-Whitney *U*-test.

Next, we examined serum concentrations of semaphorins in 14 patients treated with DAAs and 9 patients treated with PegIFNα before and 12 weeks after completion of treatment. At baseline, liver fibrosis was classified as F1/2 in 39% and F3/4 in 61% of patients. Antiviral therapy significantly improved fibrosis stage, as measured by fibroscan 12 weeks after treatment completion, with 60% of cases classified as F1/2 after therapy (Fig 3A). After treatment all patients had AST and ALT values within reference range.

Importantly, serum concentrations of SEMA3C and SEMA6D significantly decreased after DAA (4.35, IQR 3.62–5.03 to 0.64, IQR 0.28–0.96, p<0.0001, and 4.13, IQR 1.2–5.85 to 1.69, IQR 0.84–2.67, p = 0.0340, respectively) and PegIFNα therapy (3.49, IQR 2.00–5.03 to 1.46, IQR 0.96–2.1, p = 0.0083 and 3.17, IQR 1.81–4.87 to 1.23, IQR 0.59–2.19, p = 0.0244, respectively). The serum concentration of SEMA5A significantly increased after DAAs therapy (11.05, IQR 8.67–15.75 to 15.75, IQR 13.65–22.88, p = 0.0241), but there were no significant changes in patients treated with PegIFNα (Fig 3B–3D). There were no significant changes in serum concentrations of SEMA6B.

### SEMA3C and SEMA5A expression in liver tissue samples

**Fig 4:**
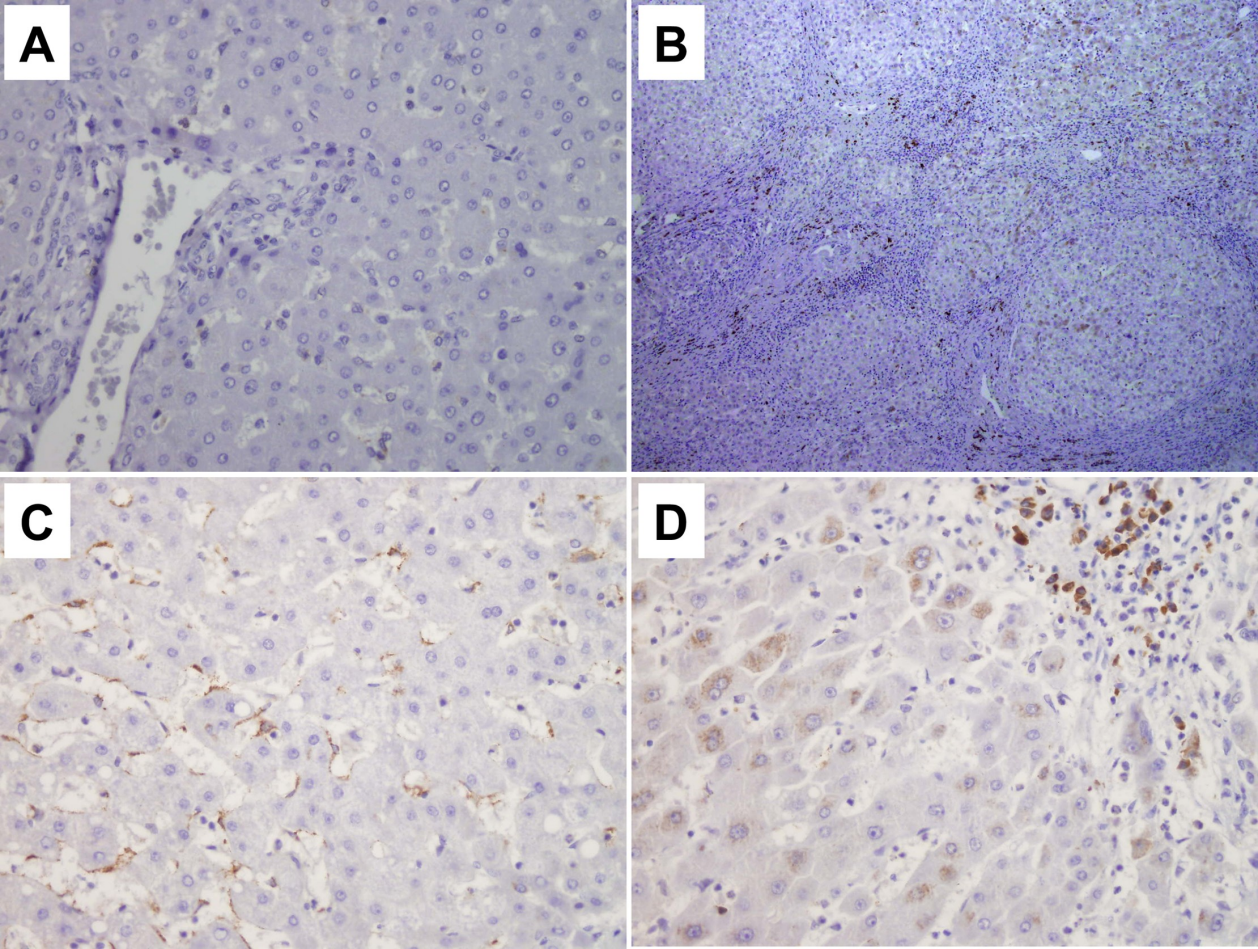
Immunohistochemical staining of SEMA3C in healthy and cirrhotic HCV liver. While there is no expression of SEMA3C markers in healthy liver tissue (**A**, magnification 40x), in cirrhotic HCV liver (**B**, magnification 10x), there is a moderate expression in endothelial cells and lymphocytes (**C,** magnification 40x) and weak in hepatocytes (**D,** magnification 40x).

**Fig 5:**
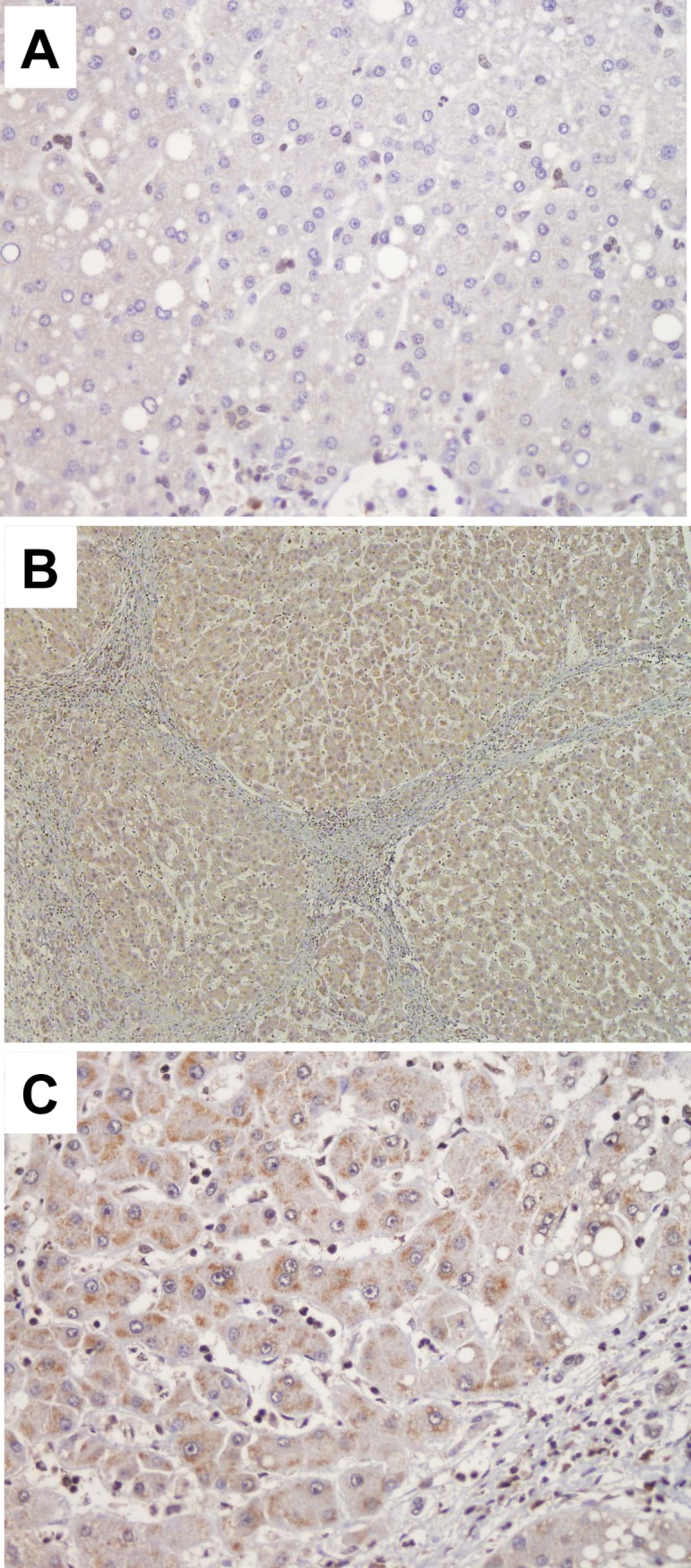
Immunohistochemical staining of SEMA5A in healthy and cirrhotic HCV liver. While there is no expression of SEMA5A markers in healthy liver tissue (**A**, magnification 40x), there is a strong expression in HCV cirrhotic livers (**B,** magnification 10x), in endothelial cells and lymphocytes and in hepatocytes (**C,** magnification 40x).

To gain further insight into tissue expression of SEMA3C and SEMA5A, we performed immunohistochemistry in cirrhotic CHC human livers in comparison to healthy donors. In healthy livers SEMA3C and SEMA5A expression was not detected by immunohistochemistry, neither in hepatocytes, nor in endothelial cells (Fig 4A and Fig 5A). By contrast, in CHC cirrhotic livers SEMA3C and SEMA5A expression was detected in hepatocytes as well in endothelial cells and lymphocytes. In HCV cirrhosis, SEMA3C showed moderate positivity in endothelial cells and lymphocytes (Fig 4C) and weak positivity in hepatocytes (Fig 4D). SEMA5A was expressed mostly in hepatocytes (Fig 5B and 5C). In addition, SEMA3C and SEMA5A were expressed in HCV HCC samples.

## Discussion

During chronic HCV infection, a sustained cytokine production drives persistent low-grade inflammation in the absence of immunocompetent Th1 responses thus allowing the constant viral replication [18]. However, the understanding of these phenomena remains unclear. The impairment of balance in the cytokine milieu modulates local T helper and Treg responses, which are unable to eliminate the infection, but drive hepatic damage and fibrogenesis [19]. Indeed, many cytokines have shown to be good predictors of interferon treatment responses [19, 20], however they failed to show the clinically relevant diagnostic accuracy in identifying patients with severe fibrosis or therapeutic application.

There is growing evidence that the signaling pathways triggered by semaphorin interactions with their receptors plexins and neuropilins influence the outcome of the immune responses; while some suppress immune cell activation and proliferation, others stimulate immune-mediated responses [21]. Here we provide the first evidence that semaphorins might play a role in HCV infection as well.

Firstly, we demonstrated that circulating levels of SEMA3A, SEMA3C, SEMA5A, SEMA6B and SEMA6D are detectable and elevated in the serum of patients with CHC. Class 3 semaphorins, specifically SEMA3A, previously shown to inhibit the T-cell activation, act as a positive regulator in the innate immune system by promoting proinflammatory cytokines production [12, 13, 22]. The importance of SEMA3A in autoimmune disorders is highlighted by the finding of reduced expression of SEMA3A in patients with rheumatoid arthritis and SLE that correlates with T-cell mediated inflammation and disease severity [21]. Less is known about the immunoregulatory role of SEMA3C, but it was shown that SEMA3C promotes the migration of dendritic cells [12, 13]. SEMA4D, activator of T- and B- cells, was shown to induce inflammation [12, 13]. SEMA5A promotes T-cell and NK cell proliferation and induces the secretion of Th17 proinflammatory cytokines that are implicated in SLE and RA disease activity [12, 14, 21]. SEMA6D is involved in the regulation of late phases of T-cell activation and generation of T-cell memory [12, 21].

Secondly, we found that semaphorin expression correlates with fibrosis stage. The role of semaphorins in fibrogenesis has not been studied, so the pathophysiological explanation of our findings is to be determined. Our data show that SEMA3C and SEMA6D significantly increase with fibrosis stage. Interestingly, pro-fibrogenic role of SEMA3C was shown in adipose tissue where SEMA3C increased the production of extracellular matrix and growth factors that are associated with the pathogenesis of fibrosis [23]. Recently, class III semaphorins were shown to induce the migration and invasive capacity of fibroblast-like synoviocytes in the model of rheumatoid arthritis [24]. SEMA6D activates vascular endothelial growth factor-receptor 2 implicated in liver fibrinogenesis [25]. We found that serum concentration of SEMA5A inversely correlates with fibrosis. So far it was shown that SEMA5A regulates angiogenesis by increasing endothelial cell proliferation, decreasing apoptosis and extracellular matrix degradation through matrix metalloproteinase [26].

Next, we have shown that SEMA3C and SEMA6D have superior accuracy to APRI and FIB-4 in predicting the presence of cirrhosis. Early and accurate assessment of the severity of liver fibrosis is essential in the management and prognosis of patients with CHC and CHB. Two most common non-invasive scores, APRI and FIB-4, that don’t require extra laboratory testing, are limited in prediction of significant fibrosis with relatively low sensitivity (60% and 75%, respectively) and specificity (65% and 60%, respectively) [27].

Additionally, the concentrations of SEMA3C and SEMA6D decrease following treatment with antiviral drugs (DAA) or a combination of immunomodulatory and antiviral drugs (PegIFNα and ribavirin). These results show that successful treatment resulting in viral eradication reduces the intensity of semaphorin synthesis. Additionally, they suggest that HCV infection itself, particularly in liver hepatocytes as shown by immunohistochemistry, might represent a key stimulus for semaphorin synthesis in chronic infection.

Although our results are limited by the relatively small number of patients, we provide the first evidence of the role of semaphorins in HCV infection. Due to ethical issues we assessed the stage of liver fibrosis by non-invasive technics only, as recommended and described in current international guidelines.

In conclusion, we demonstrated that circulating levels of SEMA3A, SEMA3C, SEMA5A, SEMA6B and SEMA6D are detectable and elevated in the serum of patients with CHC. Based on the correlation with the biochemical and fibroelastography degree of the liver damage, SEMA3C, SEMA5A and SEMA6D can be considered markers of liver injury in CHC and are superior to APRI and FIB-4 in predicting the presence of cirrhosis. The regulation of semaphorins and their receptors could be a potential future therapeutic target to decrease liver inflammation and fibrogenesis in chronic viral hepatitis.
